# Acute Cystitis Symptom Score (ACSS): Clinical Validation of the Italian Version

**DOI:** 10.3390/antibiotics9030104

**Published:** 2020-03-02

**Authors:** Tommaso Di Vico, Riccardo Morganti, Tommaso Cai, Kurt G. Naber, Florian M.E. Wagenlehner, Adrian Pilatz, Jakhongir Alidjanov, Girolamo Morelli, Riccardo Bartoletti

**Affiliations:** 1 Department of Translational Research and New Technologies, University of Pisa, 56126 Pisa, Italy; 2 SOD Clinical Trial Statistical Support, Azienda Ospedaliero Universitaria Pisana, 56126 Pisa, Italy; 3 Urology Unit, S. Chiara Regional Hospital, 38122 Trento, Italy; 4 Department of Urology, Technical University of Munich, 80333 Munich, Germany; 5 Clinic of Urology, Pediatric Urology and Andrology, Justus Liebig University Giessen, 35390 Giessen, Germany; 6 Department of Critical Medicine, University of Pisa, 56126 Pisa, Italy

**Keywords:** cystitis, female, urinary tract infection, pain, questionnaire, acute cystitis symptom score, ACSS

## Abstract

Acute Cystitis Symptom Score (ACSS) is an 18-item self-reporting questionnaire for clinical diagnosis and follow-up of acute uncomplicated cystitis (AUC) in women. The ACSS, originally developed in Uzbek and Russian languages, is now available in several languages. The purpose of the study was to validate the ACSS questionnaire in the Italian language. Linguistic validation was carried out according to Linguistic Validation Manual for Patient-Reported Outcomes Instruments guidelines. Clinical validation was carried out by enrolling one hundred Italian-speaking women. All women were asked to fill in the ACSS questionnaire during their medical visit. Fifty-four women, median age 36 (Inter Quartile Range 28–49), were diagnosed with AUC, while 46 women, median age 38 (IQR 29–45), were enrolled as the control group attending the hospital’s fertility center for couples. The most frequently isolated pathogen in AUC was *Escherichia coli* (40; 74.0%) followed by *Enterococcus faecalis* (7; 13.0%) and *Staphylococcus saprophyticus* (3; 5.6%). Receiver operating characteristic (ROC) curve analysis performed at the first diagnostic visit on a typical symptoms domain cut-off score of 6 revealed a sensitivity of 92.5% and specificity of 97.8%. The Italian version of the ACSS has proved to be a reliable tool with a high accuracy in diagnosis and follow-up in women with AUC. The ACSS may also be useful for clinical and epidemiological studies.

## 1. Introduction

Acute uncomplicated cystitis (AUC) is one of the most common and widespread pathological conditions among women of all ages, with a relevant impact on social costs and quality of life [1]. Although AUC is a benign disease, recurrent episodes are associated with reduction in quality of life, everyday activities, such as social and familiar relationships, leisure time and physical activities, working ability, and psychosexual disorders [2].

The guidelines on urological infections of the European Association of Urology (EAU) do not consider urinalysis or other microbiological investigations as necessary in the case of patients with AUC at their first episode [3]. As a consequence, in Italy, many general practitioners’ prescriptions are based just on a telephone conversation about the patients’ complaints and symptoms [2,4]. Antibiotic stewardship programs aim to coordinate strategies to enhance health outcomes, decrease the use of broad-spectrum antibiotics, and slow down the increase of antimicrobial resistance [5,6]. For these reasons, usage of broad-spectrum antibiotics should be limited only to individual cases. On the other hand, several non-infectious diseases, such as painful bladder syndrome, urolithiasis, and overactive bladder, may also be related to the same symptoms and, in the case of recurrence after empirical antibiotic therapy, urinalysis and microbiological investigations become mandatory. However, the perception of symptoms remains of paramount importance for the diagnostic recognition of an acute cystitis episode. Different urinary symptoms have been described by patients who frequently experience recurrent episodes of cystitis. There are, however, no questionnaires available to gauge the severity of symptoms and their interference with daily activities and to monitor treatment efficacy.

The Acute Cystitis Symptom Score (ACSS) was developed under the hypothesis that the diagnosis of acute cystitis can be made with high probability based on the typical symptoms, such as frequency, urgency, and dysuria, in the absence of vaginal and/or urethral discharge [7]. In particular, the ACSS questionnaire was developed for: (a) detection and evaluation of the severity of acute cystitis symptoms, (b) assessing the impairment of everyday activities and quality of life caused by the symptoms, and (c) differentiation of AUC from other disorders that present with similar symptoms [8]. The Uzbek and Russian versions of the ACSS were originally created and tested in Uzbek- and Russian-speaking female populations of the Republic of Uzbekistan. Thereafter, the ACSS was translated into and validated in the German, British English, Hungarian, and Tajik languages (www.acss.world). The ACSS and its scoring system have demonstrated high values of reliability, validity, and discriminative abilities in all studies conducted in these countries. The Polish, Romanian, Ukrainian, Turkish, French, Portuguese, Spanish, American English, and traditional Chinese versions have been already linguistically validated and are under definite clinical validations [8,9,10,11,12,13]. Here, we aimed to validate the ACSS in the Italian language for clinical use and for epidemiological studies.

## 2. Materials and Methods

This study was designed as a prospective, observational cohort study in women with symptoms and a microbiological diagnosis of AUC, appropriately treated and monitored as recommended in the EAU guidelines [3]. A second cohort of women attending the hospital’s fertility center for couples and not suspected of having a urinary tract infection (UTI) was enrolled as the control group. The two cohorts of women were considered for the clinical validation of the Italian ACSS.

### 2.1. Clinical and Microbiological Considerations

In accordance with EAU guidelines on urological infections, the diagnosis of a UTI was defined according to the following parameters: patient reported symptoms, patient interview, physical examination, bed-side dip-stick urinalysis, and urine culture [3]. In addition to the urological examinations, all women included into the study were asked to fill out the final Italian version of the ACSS questionnaire (see below). All clean-catch midstream urine samples collected at room temperature were immediately taken to the laboratory under refrigerated conditions and analyzed. All urine samples were analyzed for common uropathogenic bacteria and yeasts and aliquoted for DNA extraction and polymerase chain reaction testing for Chlamydia trachomatis, Neisseria gonorrhoeae, and urogenital Mycoplasma. For microbiological diagnosis, a colony count of ≥10^5^ CFU/mL was considered the cutoff for significant bacterial growth. Appropriate therapy for those women diagnosed with AUC was prescribed according to EAU guidelines [3].

### 2.2. The ACSS Questionaire

The ACSS questionnaire was developed and originally validated in the Uzbek and Russian languages as a simple and self-reported questionnaire that is helpful for the diagnosis of AUC in women [7,9]. The ACSS contains 18 questions (items) that are divided into 4 domains: 6 items regarding typical acute cystitis symptoms (“Typical” domain), 4 items for differential diagnosis (“Differential” domain), 3 items on quality of life (‘QoL’ domain), and 5 additional questions regarding other relevant circumstances, such as menstruation and pregnancy (“Additional” domain). The first 3 domains are designed and scored on a Likert-type scale in order to measure the severity of symptoms, while the items of the last domain are dichotomously designed by only requiring simple ‘Yes/No’ answers. The Likert-type scale included four classes of symptom severity according to the following terms: no symptoms (0), mild (1), moderate (2), severe (3) [14].

### 2.3. Translation Process

**Figure 1:**
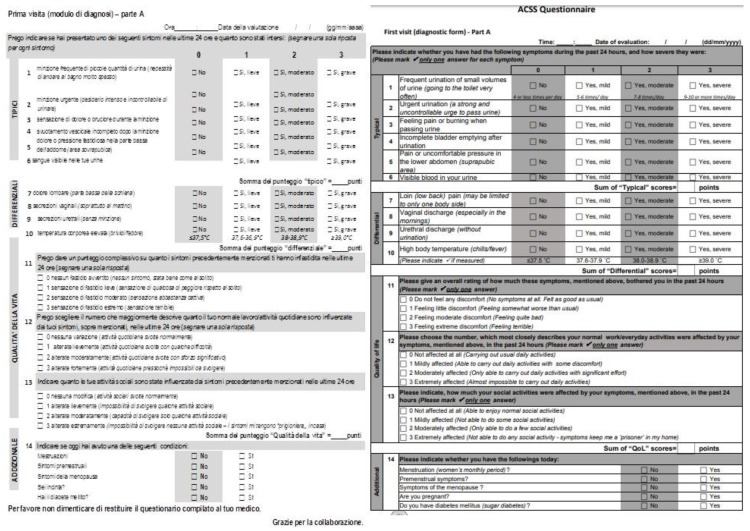
ACSS Italian and UK English version [11].

The translation and linguistic validation of the Italian version of the ACSS was performed in accordance with the Linguistic Validation Manual for Patient-Reported Outcomes (PRO) Instruments guidelines [15]. Starting from the validated UK (British) English version of the ACSS [11], two forward translations into the Italian language were produced by two professional independent translators. After a consultative meeting between the two independent translators and the local project manager, the “first consensus version” was obtained. Then, the “first consensus version” was back-translated to UK English language by an independent British English native speaker translator. This version was compared with the validated UK (British) English version of the questionnaire in order to detect, discuss, and eliminate any relevant differences. Then this “corrected provisional version” of the questionnaire was used for a cognitive assessment test carried out on 10 Italian-speaking women with different educational levels. All comments of the patients were then discussed between the translators and the local project team and the final version of the questionnaire was created (Figure 1).

### 2.4. Recruitment and Validation of the Cohort Population

All female patients (aged 18 years and older) with clinical and/or microbiological diagnosis of AUC, attending a single Italian center from November 2018 to February 2019 were enrolled as patients in our validation cohort. Patients with known diabetes mellitus or in treatment with pharmacological or herbal compounds were excluded from patient selection. In addition, women selected consecutively among those attending the hospital’s fertility center for couples and not suspected for AUC were enrolled as controls. The double-blind technique was applied for the respondents’ allocation into the groups. One of the urologists had access to the case histories and the results of the respondents’ clinical and laboratory investigations, but he was blinded to the results of the questionnaire survey. A second urologist was blinded to all of the results of the respondents’ investigations, apart from the ACSS test results. Another urologist compared the two independent diagnoses and, when their opinions coincided, true negative or true positive diagnoses were marked and the respondents were divided into two groups accordingly: the control group (Controls) and the acute cystitis group (Patients). Urinalysis with dip sticks and urine culture were performed in all subjects selected before their study enrolment. The ACSS questionnaire was administered prior (t0) and after treatment (t1) as required.

### 2.5. Data Analysis and Ethical Consideration

The categorical data were described with absolute frequency and the quantitative data with the median (interquartile range, IQR). A Kolmogorov–Smirnov test was used to asses if the quantitative data are normally distributed [16]. A Mann–Whitney two-sided test (Mann-Whitney’s U test) was used to compare the quantitative variables at baseline (frequency, urgency, painful urination, incomplete emptying, suprapubic pain, and hematuria) between patients and controls [17]. Receiver operating characteristic analysis (ROC analysis), using a non-parametric test, was performed to determine the best cut-off of the typical domain total score at baseline. Sensitivity and specificity were also determined. The Wilcoxon two-sided test was used to evaluate the variation of the typical domain’s variables over time [18]. Internal reliability was assessed with Spearman’s rank correlation coefficient (Spearman’s rho) [19]. All of the data obtained were analyzed using Statistical Package for the Social Sciences (IBM SPSS Statistics for Windows, Version 25.0).

### 2.6. Ethical Approval

Ethical approval was waived by the local Ethics Committee in view of the observational nature of the study and all the procedures being performed were part of the routine care. Informed consent was obtained from all individual participants included in the study.

## 3. Results

### 3.1. Demographics

Fifty-four female patients diagnosed with acute bacterial, uncomplicated cystitis were consecutively selected in our hospital’s outpatient office, whilst 46 women attending the hospital’s fertility center for couples and not suspected for UTI were considered as the control group. None of the control group patients had pyuria or nitrites at the urinary dipstick test, but several women had urinary symptoms due to other urological disorders, such as overactive bladder, urinary stones, interstitial cystitis, or obstructive cystocele. No bladder cancer or other neoplasms were found in both groups (Table 1).

### 3.2. Microbiological Findings

Fifty-four positive urine cultures were collected during the study in patients with AUC. Among these, the most common pathogens isolated were *Escherichia coli* (40) followed *by Enterococcus faecalis* (7), *Staphylococcus saprophyticus* (3), *Proteus mirabilis* (2), *Pseudomonas aeruginosa* (1), and *Klebsiella pneumoniae* (1). We observed that patients with *Enterococcus faecalis* infection had milder symptoms than patients with other microorganism infections.

### 3.3. Validity and Reliability

**Figure 2:**
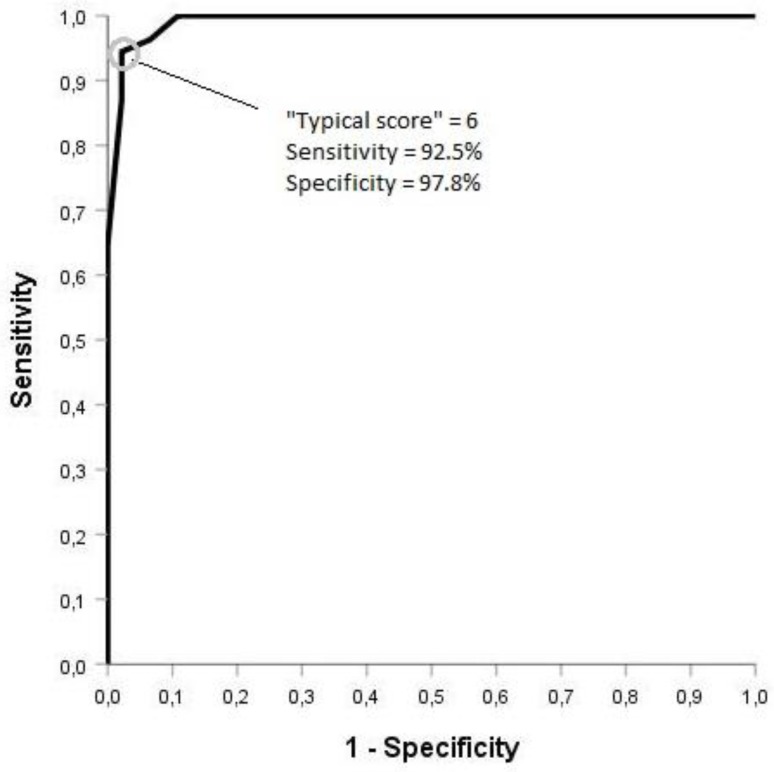
ROC analysis on the “Typical Domain” score at baseline.

**Figure 3:**
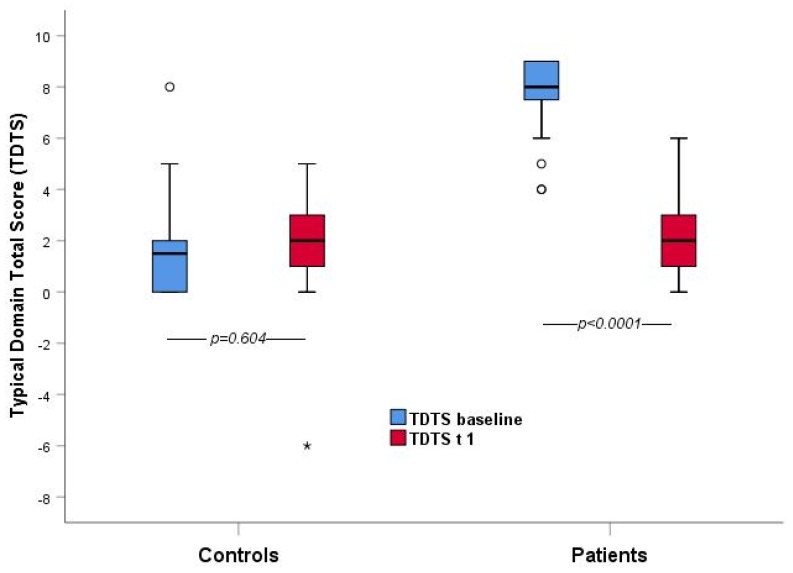
Comparison of “Typical Domain” total score between patients and controls at the baseline and T1 (the average interval between the two visits was 10 days).

Spearman’s rho, calculated between the items of the typical domain was 0.276 (*p* = 0.044) and, at the same time, Pearson’ r was 0.3 (*p* = 0.027). ROC analysis was performed on the “Typical Domain” score in order to evaluate the questionnaire’s discriminative ability. For the prediction of acute cystitis, a cut-off score of 5.5 of the typical domain showed the best balanced sensitivity (94%) and specificity (98%), respectively (Figure 2 and Figure 3). Since no individual patient can have a total score of 6, we also calculated these parameters for total scores of 5 (sensitivity 95.3%, specificity 95.7%) and 6 (sensitivity 92.5%, specificity 97.8%).

### 3.4. Comparative Analysis

Comparative analysis showed statistically significant differences between the patient’s and control’s baseline scores in each item belonging to the typical, differential, and quality of life domains at *p* < 0.05 (except for “feeling feverish” *p* = 0.35). Statistical significance for the domain mentioned above was also found for the difference between the first and second visit in female patients diagnosed with AUC (except for the item “feeling feverish” *p* = 0.356).

## 4. Discussion

### 4.1. Main Findings

This Italian version of the ACSS has proven to be useful to diagnose AUC in women due to the statistically significant correlations between patients with AUC and the controls without AUC in the typical domain scores and the results of the urinalyses. Although urinary symptoms may be typical for acute cystitis, they are, however, not specific because they are also found in patients with other urological disorders. [20,21]. Therefore, not only their presence but also their severity need to be considered. The distinctiveness of the ACSS questionnaire is the assessment of the severity of symptoms, their effect on quality of life, and the differentiation of cystitis from other urogenital disorders.

Some of the women included in the control group reported urinary symptoms, such as mild or moderate frequency, urgency, and painful urination, but no significant bacteriuria, as defined above, was found in the urine culture test. The ACSS questionnaire was also able to differentiate subjects with AUC from patients with painful bladder syndrome/interstitial cystitis, vulvodynia, overactive bladder, or other non-bacterial diseases in all cases, thus demonstrating its efficient clinical applicability. In a retrospective study on 442 patients Sun and Harlow [21] demonstrated that 44.8% of patients with vulvodynia had a history of UTI.

### 4.2. Results in the Context of Previous Studies and Clinical Applicability

The Italian version of the ACSS showed a sensitivity of 94% and 92.5% and a specificity of 98% and 97.8% at a total score for the “Typical” domain of 5.5 and 6, respectively, in line with the validation in other languages. The Hungarian ACSS version, for example, showed a sensitivity of 90% and a specificity of 97% at a total score of 6 for the “Typical” domain [12]. The results are also quite similar to the original Uzbek and Russian versions of the ACSS [8,9].

In the era of antibiotic crises and bacterial antibiotic resistance, an easy and reliable tool for improving diagnostic accuracy and antibiotic prescription is urgently required. All international guidelines on urological infections recommend the use of empirical treatment on the basis of local epidemiology of bacterial resistance and antibiotic stewardship programs. The first and the most important step, however, is the correct diagnosis of AUC. EAU guidelines suggest performing a correct assessment of all patient’s symptom and risk factors, but no validated and self-administered questionnaire for clinical diagnosis was available so far in the Italian language. Such a questionnaire could also be used for self-diagnosis and self-assessment of patient-reported outcomes to compare the efficacy of different treatment modalities, such as antibiotic and non-antibiotic therapies. In 2016, Alidjanov et al. developed and validated the first version of the Acute Cystitis Symptom Score (ACSS) in Uzbekistan in the Uzbek and Russian languages [7,8]. This questionnaire has already been translated and clinically validated in Russian, British English, German, Hungarian, and Tajik languages, with hundreds of women enrolled [14]. The linguistically- and clinically-validated Italian version of the ACSS is now also available for the diagnosis and management of women affected by AUC and could also be used in everyday clinical practice for improving the adherence to the principles of antibiotic stewardship as well as for clinical and epidemiological trials.

### 4.3. Study Limitations

This study showed few limitations to take into account. One is the single-center design of the study. Even if performed in a single center, the study is representative of the Italian women population due to the high variability in subjects born or permanently resident in other areas of the country and having moved by chance to this touristic city.

Another limitation may be seen in the subjective response when using a self-administered questionnaire due to individual interpretation of the questions asked. To minimize this possible limitation, the Italian version of the ACSS was not only forward and backward translated from English to Italian by two independent professionals, according to international rules (see certifications in the supplementary materials), but was finally established after an intensive cognitive assessment procedure interviewing 10 women with Italian as their mother language and with different educational levels.

A third limitation may be seen in the difficult clinical differentiation from other urological disorders, such as overactive bladder, urinary stones, interstitial cystitis, or obstructive cystocele, that report similar symptoms, which only could be differentiated from AUC by a positive urine culture [21,22]. However, the main clinical differences, besides a positive urine culture, are the acute onset and severity of typical symptoms in the case of AUC compared to the other urological disorders.

## 5. Conclusions

The ACSS questionnaire has proven to be a simple, easy, and affordable diagnostic tool that can be used in general medical practice and research, such as in well-designed clinical and epidemiological studies [23], for the diagnosis of AUC and as a patient-reported outcome measure.

## Figures and Tables

**Table 1:** Clinical features and comparison of the factors between groups at baseline (t0).

		Patients (*n* = 54)		Controls (*n* = 46)		
Age: Median (IQR)		38 (28; 49)		36.5 (25; 44)		
	Likert Scale		Median (IQR)		Median (IQR)	*p*-value
Frequency (n)	0 (no)	0	1 (1; 2)	33	0 (0; 1)	<0.0001
	1 (mild)	17		11		
	2 (moderate)	22		2		
	3 (severe)	15		0		
Urgency (n)	0 (no)	0	2 (1; 2)	30	0 (0; 1)	<0.0001
	1 (mild)	16		10		
	2 (moderate)	28		6		
	3 (severe)	10		0		
Painful Urination (n)	0 (no)	7	1 (1; 2)	33	0 (0; 1)	<0.0001
	1 (mild)	24		12		
	2 (moderate)	14		1		
	3 (severe)	9		0		
Incomplete Emptying (n)	0 (no)	6	1 (1; 1)	35	0 (0; 0)	<0.0001
	1 (mild)	29		10		
	2 (moderate)	13		1		
	3 (severe)	6		0		
Soprapubic Pain (n)	0 (no)	6	1 (1; 2)	38	0 (0; 0)	<0.0001
	1 (mild)	26		7		
	2 (moderate)	16		1		
	3 (severe)	6		0		
Hematuria (n)	0 (no)	6	1 (1; 1)	43	0 (0; 0)	<0.0001
	1 (mild)	32		2		
	2 (moderate)	15		1		
	3 (severe)	16		0		
Flank Pain (n)	0 (no)	25	1 (0; 2)	44	0 (0; 0)	<0.0001
	1 (mild)	13		2		
	2 (moderate)	16		0		
	3 (severe)	0		0		
Vaginal Discharge (n)	0 (no)	19	1 (0; 2)	46	0 (0; 0)	0.0003
	1 (mild)	16		0		
	2 (moderate)	18		0		
	3 (severe)	0		0		
Urethral Discharge (n)	0 (no)	19	1 (0; 1)	38	0 (0; 0)	<0.0001
	1 (mild)	24		7		
	2 (moderate)	9		1		
	3 (severe)	2		0		
Feeling Feverish (n)	0 (no)	20	1 (0; 1)	46	0 (0; 0)	<0.0001
	1 (mild)	23		0		
	2 (moderate)	11		0		
	3 (severe)	0		0		
General Discomfort (n)	0 (no)	1	2 (1; 3)	40	0 (0; 0)	<0.0001
	1 (mild)	14		6		
	2 (moderate)	21		0		
	3 (severe)	18		0		
Impact on Everyday Activity (n)	0 (no)	6	2 (1; 2)	42	0 (0; 0)	<0.0001
	1 (mild)	16		4		
	2 (moderate)	23		0		
	3 (severe)	9		0		
Impact on Social Life (n)	0 (no)	1	2 (1; 3)	40	0 (0; 0)	<0.0001
	1 (mild)	15		5		
	2 (moderate)	18		1		
	3 (severe)	20		0		

The median (IQR) age of the Controls and Patients was 36 (28–49) and 38 (29–45) years, respectively.

## Supplementary Materials

Supplementary Materials are available online at https://www.mdpi.com/2079-6382/9/3/104/s1.

## Copyright and Translations of the ACSS in Other Languages

The ACSS is copyrighted by the Certificate of Deposit of Intellectual Property in Fundamental Library of Academy of Sciences of the Republic of Uzbekistan, Tashkent (Registration number 2463; 26 August 2015) and the Certificate of the International Online Copyright Office, European Depository, Berlin, Germany (Nr. EU-01-000764; 21 October 2015). The rightsholders are Jakhongir Fatikhovich Alidjanov (Uzbekistan), Ozoda Takhirovna Alidjanova (Uzbekistan), Adrian Martin Erich Pilatz (Germany), Kurt Guenther Naber (Germany), Florian Martin Erich Wagenlehner (Germany).
